# Identification, Expression Patterns, and Functional Characterization of Chemosensory Proteins in *Dendroctonus armandi* (Coleoptera: Curculionidae: Scolytinae)

**DOI:** 10.3389/fphys.2018.00291

**Published:** 2018-03-27

**Authors:** Zhumei Li, Lulu Dai, Honglong Chu, Danyang Fu, Yaya Sun, Hui Chen

**Affiliations:** ^1^College of Forestry, Northwest A&F University, Yangling, China; ^2^College of Forestry and Landscape Architecture, South China Agricultural University, Guangzhou, China; ^3^Center for Yunnan Plateau Biological Resources Protection and Utilization, College of Biological Resource and Food Engineering, Qujing Normal University, Qujing, China

**Keywords:** chemosensory proteins, *Dendroctonus armandi*, olfaction, semiochemicals, fluorescence binding assays, RNAi, EAG

## Abstract

The Chinese white pine beetle, *Dendroctonus armandi* Tsai and Li (Coleoptera: Curculionidae: Scolytinae), is a serious pest of coniferous forests in China. Thus, there is considerable interest in developing eco-friendly pest-control methods, with the use of semiochemicals as a distinct possibility. Olfaction is extremely important for fitness of *D. armandi* because it is the primary mechanism through which the insect locates hosts and mates. Thus, here we characterized nine full-length genes encoding chemosensory proteins (CSPs) from *D. armandi*. The genes were ubiquitously and multiply expressed across different developmental stages and adult tissues, indicating various roles in developmental metamorphosis, olfaction, and gustation. Ligand-binding assays implied that DarmCSP2 may be the carrier of *D. armandi* pheromones and various plant host volatiles. These volatiles were identified through RNA interference of *DarmCSP2* as: (+)-α-pinene, (+)-β-pinene, (−)-β-pinene, (+)-camphene, (+)-3-carene, and myrcene. The systematic chemosensory functional analysis of DarmCSP2 in this study clarified the molecular mechanisms underlying *D. armandi* olfaction and provided a theoretical foundation for eco-friendly pest control.

## Introduction

Chemoreception (olfaction and gustation) is an indispensable biological process for many insect species (Sánchez-Gracia et al., 2009), playing a vital role in detecting the specific semiochemicals emitted by host plants or conspecifics (Yoshizawa et al., 2011). To accurately perceive such semiochemicals, insects have evolved a sophisticated, sensitive, and specific chemosensory system (Karg and Suckling, 1999; Field et al., 2000). Numerous olfactory protein groups have been identified in the insect chemosensory system, with wide-ranging functions that include locating food sources, recognizing conspecifics and predators, as well as identifying oviposition sites; these include odorant-binding proteins (OBPs), chemosensory proteins (CSPs), olfactory receptors (ORs), gustatory receptor (GRs), and odorant degrading enzymes (ODEs) (Sánchez-Gracia et al., 2009; Leal, 2013). While CSPs and OBPs have similar function, they share no sequence similarity (Pelosi et al., 2005; Gong et al., 2007). The special tertiary structure of CSPs with hydrophilic surface and hydrophobic cavity allow them to distinguish, capture, and bind hydrophobic chemicals from external environments to ORs or GRs (Pelosi et al., 2005, 2017; Gong et al., 2007; Sánchez-Gracia et al., 2009; Liu et al., 2012; Leal, 2013).

Unsurprisingly, given their critical functions, chemosensory proteins are widespread and have been isolated from multiple insect orders (McKenna et al., 1994; Angeli et al., 1999; Robertson et al., 1999; Marchese et al., 2000; Forêt et al., 2007; Andersson et al., 2013; Li et al., 2013; Yang et al., 2014; He et al., 2017). In insects of both sexes, CSPs are broadly expressed throughout development (Stathopoulos et al., 2002; Wanner et al., 2005; Qiao et al., 2013; Yang et al., 2014; Li et al., 2016) and across tissue types, including antennae, heads, thoraxes, abdomens, proboscis, eyes, legs, wings, pheromone glands, and reproductive organs (Nomura et al., 1992; Field et al., 2000; Nagnan-Le Meillour et al., 2000; Ban et al., 2003; Gu et al., 2013; Li et al., 2013; Zhou et al., 2013; Zhu et al., 2016; Wang et al., 2017). Fluorescence competitive binding assays have indicated that CSPs bind to a wide range of compounds, such as plant volatiles, insect pheromones (Briand et al., 2002; Li et al., 2015), cuticular hydrocarbons and lipids (Ozaki et al., 2005; González et al., 2009), as well as visual pigments (Zhu et al., 2016). These sophisticated expression profiles and binding ability suggest that the role of CSPs is complex, spanning from chemoreception to other functions in development, vision, nutrition, reproduction, and regeneration (Nomura et al., 1992; Briand et al., 2002; Wanner et al., 2005; Li et al., 2015; Zhu et al., 2016; Pelosi et al., 2017).

Clarifying the mechanisms underlying CSP function not only improves our understanding of insect biology but also has strong practical value for developing eco-friendly pest control. Because many insect pests are so dependent on olfaction to find hosts and mates, damage to olfactory systems or targeted release of host volatiles or pheromones to alter insect behavior should be effective control methods that do not negatively impact the surrounding ecosystem. For example, the Chinese white pine beetle, *Dendroctonus armandi* Tsai and Li (Coleoptera: Curculionidae: Scolytinae), uses aggregation pheromones to coordinate mass attacks on host trees, whereas odorants from host and non-host trees modulate pheromone response (Zhang and Schlyter, 2004; Erbilgin et al., 2007; Andersson et al., 2010, 2013). The beetle responds to volatiles emitted from both host and non-host plants, as well as insect pheromones (Zhang et al., 2010; Xie and Lv, 2012; Chen et al., 2015; Zhao et al., 2017a,b). This serious pest of coniferous forests in China's Qinling and Bashan Mountains primarily attacks healthy Chinese white pine (*Pinus armandi* Fr.), residing in the phloem across all life stages except for a brief dispersal period to mate and find new hosts (Ren and Dang, 1959; Cai, 1980; Chen and Tang, 2007). In particular, *D. armandi* infestation has damaged large swathes of *P. armandi* forests, incurring heavy economic losses and serious ecological destruction (Chen and Tang, 2007; Xie and Lv, 2012). There is an urgent need to develop effective and eco-friendly *D. armandi* control, with olfaction-related methods being an attractive option. However, we currently know very little about the molecular mechanisms underlying olfactory perception in this species.

Therefore, in this study, we combined molecular and physiological methods to investigate the relationship between CSP and olfactory behavior in *D. armandi*. We identified *CSP* genes from *D. armandi* (*DarmCSPs*), and assessed their tissue and developmental expression profiles. Selected *DarmCSP*s were expressed and their binding affinity to semiochemicals were tested. Finally, we examined how DarmCSP affected *D. armandi* olfaction and ascertained the specific semiochemicals that bind these proteins in adult beetles.

## Materials and methods

### Insect collection

Larvae and pupae of *D. armandi* were collected from the bark of infested *P. armandi* trees at the Huoditang Experimental Forest Station of Northwest A&F University, located on the southern slope of the Qinling Mountains (33°18′N, 108°21′E) in Shaanxi, China. Logging slash of infested *P. armandi* was moved from the sample plot to a greenhouse, where adult beetles were collected as they emerged and then kept at 4°C on moist paper. Adults were sexed based on external genitalia and male-specific auditory cues (Dai et al., 2014; Zhao et al., 2017a).

### Reagents

Contech Enterprises (Delta, BC, Canada) provided (±)-exo-brevicomin and (±)-frontalin. Bedoukian Research (Danbury, CT, USA) provided (–)-trans-verbenol. Finally, (1S)-(–)-verbenone, HPLC-grade hexane, 1-hexanol, and methanol, as well as 10 host volatiles of *D. armandi* were purchased from Sigma-Aldrich.

### Identification of *D. armandi CSP* genes

#### RNA isolation and cDNA synthesis

Total RNA for RT-PCR was isolated from larvae, pupae, and adults of both sexes using the UNIQ-10 Column TRIzol Total RNA Isolation Kit (Sangong, Shanghai, China), following manufacturer protocol. RNA integrity was verified with 1.0% agarose gels electrophoresis and quantified with spectrophotometry in a NanoDrop 2000 (Thermo, Pennsylvania, USA). Total RNA from the three developmental stages were mixed for cDNA synthesis with the PrimeScript™ RT Reagent Kit with gDNA Eraser (TaKaRa, Dalian, China), following manufacturer protocol. Single-stranded 5′and 3′ RACE-ready cDNA was synthesized from mixed RNA (1 μg) using a SMARTer RACE cDNA Amplification Kit (Clontech, CA, USA), following manufacturer protocol, then stored at −20°C until use.

#### Gene amplification and cloning

Synthesized cDNA was used as a template in PCR reactions. Degenerate and specific primers (Table S1) were designed in Primer Premier 5.0, based on CSP sequences of other insects from NCBI (http://www.ncbi.nlm.nih.gov/). PCR amplifications were performed in a C1000 thermocycler (Bio-Rad, CA, USA), under the following conditions: initial denaturation for 3 min at 95°C; followed by 30 cycles of 30 s at 95°C, 30 s at 50–60°C, 1 min at 72°C; and then a final extension for 10 min at 72°C. The 20 μL reaction mixture contained 1 μL cDNA (1:10 dilution), 0.25 μM of each primer, and 2 × EcoTaq PCR SuperMix (TransGen, Beijing, China). PCR products were visualized on 1% agarose gels using 1 × 4S Red Plus Nucleic Acid Stain (Sangong, Shanghai, China) and compared with a 2 K plus DNA marker (TransGene, Beijing, China). Amplified fragments were purified using the Gel Purification Kit (Omega, GA, USA), ligated into pMD™ 18-T Vector (TaKaRa, Dalian, China), and transformed into DH5α chemically competent *Escherichia coli* cells (TransGen, Beijing, China). Transformants were selected on Amp/LB/Xgal/IPTG plates, and positive clones were PCR-analyzed using vector-specific primers (M_13_-47, M_13_-48). Lastly, bacterial solutions of positive clones were sequenced by a local biotechnology company (Augct, Beijing, China). Three independent clones were submitted to minimize potential PCR mutations. Sequences were manually edited with EditSeq of DNASTAR (https://www.dnastar.com/) to obtain inserts, which were then BLASTed against the NCBI database.

#### 5′ and 3′ RACE

Gene-specific inner and outer primers for 5′ and 3′ RACE (Table S1) were designed based on obtained sequence fragments. Touchdown PCR (annealing temperatures: 65–55°C) was performed to improve amplification specificity of the 5′-UTR and 3′-UTR sequences. The amplified products were visualized, purified, cloned, sequenced, and blasted as described in the previous section (“Gene amplification and cloning”).

#### Analysis of full-length cDNA sequences

Full-length cDNA sequences were assembled in *DNAMAN 6.0* (http://www.lynnon.com/), using sequence fragments and RACE results. To avoid chimera sequences, specific primers (Table S1) from initiation to terminator codon were designed based on complete sequences. High-fidelity PCR was performed using Phanta HS Super-Fidelity DNA Polymerase (Vazyme, Nanjing, China). Amplicons were cloned into pMD18-T and detected through sequencing and BLASTp search. Putative gene sequences were deposited in GenBank, and Accession Numbers were listed in Table 1. Open reading frames (ORFs) of full-length cDNA were obtained via ORF Finder (https://www.ncbi.nlm.nih.gov/orffinder/), and cDNA was then translated to amino acid sequences using the ExPASy Translate Tool (http://www.expasy.org/tools/dna.html), aligned in ClustalX 2.0.10 (Thompson et al., 1997), and colored in *DNAMAN6.0*. Molecular mass (kDa) and isoelectric points were determined in PROTPARAM (Gasteiger et al., 2005). DarmCSP homologs were identified with the NCBI-BlastP network server (https://blast.ncbi.nlm.nih.gov/Blast.cgi). Amino acid identity was analyzed through the construction of a homology tree in *DNAMAN6.0*. A neighbor-joining phylogenetic tree was built in MEGA 6.0 (Tamura et al., 2011), employing ClustalW with default parameters, p-distance model, pairwise gap deletion, and 1000 bootstrap replicates. The putative N-terminal signal peptide was predicted in Signal P 4.1 Server (http://www.cbs.dtu.dk/services/SignalP/).

**Table 1:** Physicochemical properties and blastp matches of putative *DarmCSP* genes.

**Gene name**	**Accession**	**Full Length**	**ORF^a^**	**Signal^b^**	**M.W^c^**		**Best Blast match in gene bank**			
	**no**.	**(bp)**	**(aa/bp)**	**Peptide**	**(KDa)**	**IP^c^**	**Species**	**Gene name**	**Accession no**.	**Identity%^d^**
*DarmCSP1*	MG637034	539	124/375	1–16	14.339	7.57	*Dendroctonus ponderosae*	*CSP1*	AGI05161.1	92
*DarmCSP2*	MG197742	583	121/366	1–18	13.941	8.76	*Dendroctonus ponderosae*	*CSP2*	AGI05172.1	93
*DarmCSP3*	MG637035	513	138/417	1–19	15.494	4.92	*Dendroctonus ponderosae*	*CSP3*	AGI05160.1	86
*DarmCSP4*	MG637036	521	125/378	1–19	14.314	8.60	*Dendroctonus ponderosae*	*CSP4*	AEE62703.1	90
*DarmCSP5*	MG637037	1008	255/768	1–18	28.038	9.48	*Dendroctonus valens*	*CSP4*	AKK25148.1	79
*DarmCSP6*	MG637041	507	130/393	1–19	14.971	9.06	*Dendroctonus ponderosae*	*CSP6*	AGI05162.1	88
*DarmCSP7*	MG637038	567	144/435	1–20	16.411	5.42	*Dendroctonus ponderosae*	*CSP7*	AEE63473.1	85
*DarmCSP8*	MG637039	523	127/384	1–17	14.734	8.33	*Dendroctonus ponderosae*	*CSP8*	AGI05164.1	96
*DarmCSP9*	MG637040	614	115/348	1–25	13.018	9.03	*Dendroctonus ponderosae*	*CSP9*	AEE61984.1	97

ORF, open reading frame; pI, isoelectric point; MW, molecular weight;

a *As predicted by ORF Finder (https://www.ncbi.nlm.nih.gov/orffinder/)*.

b *As predicted using SignalP 4.1 Server (http://www.cbs.dtu.dk/services/SignalP/)*.

c *As predicted by Protparam program*.

d *As predicted by BLAST (http://www.ncbi.nlm.nih.gov)*.

### Expression patterns of *CSP* genes across different life stages and tissues

*D. armandi* larvae were separated into two sub-stages: larvae and mature larvae (when they stop feeding). Pupae were similarly separated into two sub-stages: early pupae (newly metamorphosed from larvae) and late pupae (close to becoming adults). Adults were separated into three sub-stages: teneral (body color still light), emerged, and feeding (invading a new host) (Dai et al., 2014). Antennae, mouthparts, heads (without antennae and mouthparts), forewings, underwings, legs, thoraxes, abdomens (without pheromone glands), and pheromone glands of male and female emerged adults were dissected. Samples were collected in triplicate, frozen in liquid nitrogen immediately, and stored at −80°C until use. RNA isolation and cDNA synthesis followed previous descriptions (“RNA isolation and DNA synthesis”).

The CFX96™ Real-Time PCR Detection System (Bio-Rad, California, USA) was used for qRT-PCR, with *D. armandi* β*-actin* (accession number: KJ507199.1) and α*-tubulin* (accession number KJ507202.1) as reference genes. Specific qRT-PCR primers were designed in Beacon Designer 7.7, based on nucleotide sequences (Table S1), and their amplification efficiencies were calculated using relative standard curves with a five-fold cDNA dilution series; the efficiency values for the primers were 100 ± 5%. The sizes of the amplicons were 231 bp (β*-actin*), 218 bp (α*-Tubulin*), 193 bp (*DarmCSP1*), 95 bp (*DarmCSP2*), 208 bp (*DarmCSP3*), 229 bp (*DarmCSP4*), 229 bp (*DarmCSP5*), 120 bp (*DarmCSP6*), 183 bp (*DarmCSP7*), 132 bp (*DarmCSP8*), and 250 bp (*DarmCSP9*). Amplicons were confirmed to be of the correct size after the qRT-PCR assay via gel electrophoresis, and then sequenced by a biotechnology company (Augct, Beijing, China) to make sure that the correct amplification products were obtained. The reaction mixture (20 μL) contained 10 μL of SYBR® Premix Ex Taq™ II (Tli RNaseH Plus) (TaKaRa, Dalian, China), 2 μL of cDNA (diluted 10 times), 0.6 μL of each primer, and 6.8 μL of nuclease-free water. Template-free negative controls were included in every reaction. Thermocycling conditions were as follows: 95°C for 10 s, followed by 40 cycles of 95°C for 5 s, and 60°C for 30 s. At the end of each reaction, a melting curve analysis was performed to detect single gene-specific peaks and check for primer dimers. Three technical and three biological replicates were performed to verify reproducibility. *DarmCSPs* expression data were generated from normalizing data to the geometric average of the internal control genes (Vandesompele et al., 2002). The comparative 2^−ΔΔCt^ method was used to calculate relative mRNA levels of *DarmCSPs* (Schmittgen and Livak, 2008); resultant values were log2-transformed for analysis of variance and plotting. Expression was normalized based on the lowest expression level.

### Binding characteristics of DarmCSPs

#### *E. coli* expression and purification of DarmCSPs

To better characterize DarmCSP function, three antennae-preferential genes (*DarmCSP 1–3*) were chosen for expression in bacteria. Signal peptides were removed to generate properly folded proteins. PCR products encoding mature proteins were amplified using gene-specific primers (Table S1), cloned into pGEM-T easy vectors (Promega, Madison, USA), then excised and cloned into the bacterial expression vector pET32a(+) (Novagen, Madison, WI), between *Bam*HI and *Xho*I restriction sites. Successful cloning was verified through PCR and sequencing. Plasmids containing the correct insert were extracted and transformed into *E. coli* BL21 (DE3) competent cells. Positive clones were incubated at 37°C until absorbance = 0.6 at OD 600, and protein expression was induced with IPTG (isopropyl-β-D-1-thiogalactopyranoside) treatment (28°C for 6 h) to a final concentration of 0.5 mM. Cells were harvested via centrifugation at 12,000 × g and 4°C for 5 min, then cleaned using PBS buffer (137 mM NaCl, 2.7 mM KCl, 10 mM Na_2_HPO_4_, 2 mM KH_2_PO_4_, pH = 7.4). After resuspension in the lysis buffer (50 mM Tris-HCl, 300 mM NaCl, 25 mM Na_2_HPO_4_, pH = 8.0), cell solution was sonicated on ice for 10 min (sonication for 3 s with an interval of 5 s), then centrifuged again at 12,000 × g and 4°C for 30 min.

Recombinant proteins were purified with N-termini His tagged from the supernatant using a Ni-NTA-Sefinose Column (Sangon, Shanghai, China), and placed in a buffer (25 mM Tris-HCl, 50 mM NaCl, and 2 mM CaCl_2_, pH 7.6) for dialysis. To avoid confounding effects in fluorescence binding assays, His-tags were excised using Recombinant Enterokinase with His-tag (rEK) (Sangong, Shanghai, China), and the resultant complex was cleared through a Ni-NTA-Sefinose Column. NaCl and CaCl_2_ were removed from DarmCSPs via dialysis in 50 mM Tris-HCl buffer (pH = 7.4). Purified proteins were stored at −80°C until use. The size and purity of DarmCSPs were checked using 12% SDS-PAGE, whereas their concentration was measured with the BCA Assay Kit (Sangong, Shanghai, China).

#### Fluorescence binding assays

A Hitachi F-4500 fluorescence spectrophotometer was used to measure emission fluorescence spectra, in a right-angle configuration with a 1 cm light-path quartz cuvette. The slit width was 5 mm for both excitation and emission. DarmCSPs were dissolved to 2 μM in 50 mM Tris-HCl buffer (pH = 7.4), whereas fluorescent probe N-phenyl-1-naphthylamine (1-NPN) and all semiochemicals were dissolved in methanol with a 1 mM stock solution. To measure DarmCSP affinity with 1-NPN, 2 mL of 2 μM DarmCSP solution was titrated with 1 mM 1-NPN to a final concentration of 2–16 μM. Excitation of 1-NPN occurred at 337 nm, with the emission spectra recorded from 360 to 500 nm. Corresponding fluorescence intensity values were plotted against free 1-NPN concentration to determine DarmCSP binding constants. Bound 1-NPN concentrations were assessed based on fluorescence intensity, assuming DarmCSPs were 100% active and protein: ligand = 1:1 at saturation. The dissociation-constants curve was linearized with Scatchard plots in Prism 6.0 (GraphPad Software, CA, USA).

Ligand affinity was measured with competitive binding assays. Fourteen compounds were selected based on previous reports (Zhang et al., 2010; Xie and Lv, 2012; Chen et al., 2015; Zhao et al., 2017a,b), including 10 host volatiles and four *D. armandi* pheromones (Table 2). A mixture of 2 μM DarmCSP and 2 μM 1-NPN was titrated with each ligand to final concentrations of 2–16 μM. Corresponding fluorescence intensities were recorded from three independent measurements. Dissociation constants of competitive ligands were calculated according to IC50 values, using the equation: KD = [IC50]/(1+[1-NPN]/K_1−NPN_), where IC50 is competitive-ligand concentration at half the initial fluorescence of 1-NPN, 1-NPN is the concentration of free 1-NPN, and K_1−NPN_ is the dissociation constant of DarmCSP with 1-NPN.

**Table 2:** Fluorescence competitive binding affinities of selected components to pure DarmCSP2.

**Ligand name**	**CAS No**.	**Purity (%)**	**IC 50 (μM)**	**K_i_**
**PHEROMONE**				
(-)-trans-Verbenol	80795-83-1	95.0	4.33 ± 0.10	2.80 ± 0.07
(±)-*exo*-Brevicomin	62532-53-0	95.0	14.48 ± 0.11	9.34 ± 0.07
(1S)-(-)-Verbenone	1196-01-6	99.0	29.27 ± 1.11	18.89 ± 0.72
(±)-Frontalin	28401-39-0	95.0	26.19 ± 1.94	16.91 ± 1.26
**VOLATILES**				
(+)-α-Pinene	7785-70-8	99.0	3.60 ± 0.03	2.33 ± 0.02
(–)-α-Pinene	7785-26-4	99.0	2.53 ± 0.11	1.64 ± 0.08
(+)-β-Pinene	19902-08-0	99.0	5.90 ± 1.31	3.8 ± 0.84
(–)-β-Pinene	18172-67-3	99.0	5.93 ± 0.17	3.83 ± 0.11
(*R*)-(+)-Limonene	5989-54-8	99.9	6.36 ± 0.25	4.10 ± 0.16
(*S*)-(-)-Limonene	5989-27-5	99.0	5.51 ± 0.42	3.41 ± 0.04
(+)-3-Carene	498-15-7	99.0	3.05 ± 0.71	1.97 ± 0.46
(+)-Camphene	5794-03-6	85.0	4.12 ± 0.33	2.66 ± 0.21
(R)-(-)-*a*-Phellandrene	4221-98-1	95.0	8.77 ± 0.24	5.66 ± 0.16
Myrcene	123-35-3	99.0	10.78 ± 0.20	6.96 ± 0.13

#### Structural model of DarmCSP2

The predicted 3D structure of DarmCSP2 was generated via homology modeling in SWISS-MODEL (https://swissmodel.expasy.org/) with default parameters (Guex et al., 2009), with the solution structure of *Schistocerca gregaria* CSP4 (Tomaselli et al., 2006) as a template (identity: 44.33%). The model was rendered in PyMol (http://www.pymol.org/). A multiple protein sequence alignment was created with ClustalX 2.0.10 (Thompson et al., 1997) and colored using ESPript (http://espript.ibcp.fr/ESPript/cgi-bin/ESPript.cgi) (Robert and Gouet, 2014).

### RNA interference of *DarmCSP2*

#### Insect treatment and qRT-PCR

As further verification of DarmCSP2 biological function, *D. armandi* adults were injected with gene-specific double-stranded RNA (dsRNA) for RNA interference (RNAi). Two pairs of special primers (T7DarmCSP2F/DarmCSP2R and DarmCSP2F/T7DarmCSP2R) were designed for dsRNA synthesis through the addition of T7 polymerase recognition region (5′-taatacgactcactatagg-3′) at the 5′ ends (Table S1). The verified pMD18-T plasmid containing *DarmCSP2* acted as a template for two high-fidelity PCRs using Phanta HS Super-Fidelity DNA Polymerase (Vazyme, Nanjing, China). Resultant cDNA, flanked by T7 polymerase promoter sequences, were electrophoresed on a 1% agarose gel and purified with the Gel Purification Kit (Omega, GA, USA). Purified amplicons were used as templates to synthesize dsRNA with T7 RiboMAX Express RNAi (Promega, USA), following manufacturer protocol. *DarmCSP2*-dsRNA (hereafter dsCSP2) integrity was checked via 1% agarose gel electrophoresis. Finally, dsCSP2 was then quantified in NanoDrop 2000 (Thermo, Pennsylvania, USA), diluted to 1000 ng/μL in DEPC water, and stored at −80°C.

Freshly and synchronously emerged adults were anesthetized on glass petri dishes placed for 30 min in an ice bath, before injection with 0.2 μL dsCSP2 into the hemocoel at the suture under the hindleg. Injections used Hamilton Microliter™ syringes (700 series, RN) with 32G sharp-point needles (Hamilton, Switzerland). Controls were either injected with 0.2 μL DEPC-treated water or not injected. Subjects were then transferred onto wet filter paper placed in clear glass petri dishes for continuous culture (at 20 ± 1°C, 50% humidity). Three males and three females were removed at different time intervals (12, 24, and 48 h) from each treatment group for storage at −80°C until qRT-PCR analysis. Three replicates were performed per treatment group (non-injected, water-injected, dsCSP2-injected). RNA isolation, cDNA synthesis, and qRT-PCR procedure followed methods described above (“RNA isolation and cDNA synthesis” and “Expression patterns of *CSP* genes across different life stages and tissues”).

#### Electroantennogram analysis

Electroantennograms (EAG) were used to detect RNAi efficiency of dsCSP2 and determine DarmCSP2 function in binding semiochemicals. Methods were modified from a previous study (Zhang et al., 2010). Subject beetles were chosen based on post-RNAi qRT-PCR and anesthetized in an ice bath. Antennae were carefully excised at their base with a scalpel and immediately connected between two electrode holders using Spectra 360 electrode gel.

Semiochemicals were selected based on the results of fluorescence binding assays and dissolved to 10 μg/μL in hexane. Hexane alone acted as a blank control and 1 μg/μL 1-hexanol was used as a standard to normalize all EAG recordings (Zhang et al., 2010). Semiochemical solutions (20 μL) were loaded onto filter paper strips (5 × 30 mm) and then transferred into a Pasteur pipette. The pipette tip was then inserted into a small hole in the wall of a steel tube (15 mm diameter × 15 cm length). The tube was connected to an air stimulus controller (CS-05b Syntech, the Netherlands) for constant humidified airflow delivery at a rate of 40 mL/min. The open end of the tube was positioned 1 cm before an antenna affixed between two electrode holders.

To stimulate the antenna, semiochemical-containing air was introduced through the pipette into the main air flow at a rate of 20 mL/min for 0.2 s. Each stimulus was separated by at least a 1 min interval to ensure complete antenna recovery. Signals were recorded using an IDAC-2 unit plus amplifier and Syntech EAG 2000 (Syntech, Netherlands). The control and standard were tested before and after every semiochemical solution. Antennae from12 individuals (six males and six females) were tested, with three replicates per antenna. To calculate EAG values, mean responses of the solvent control before and after exposure were subtracted from mean sample responses, then converted to a percentage of the accompanying standard (Zhang et al., 2010).

### Statistical analysis

Data from qRT-PCR and EAG were analyzed in SPSS Statistics 19.0 (IBM, Chicago, USA). Significant between-treatment differences in *DarmCSP* mRNA levels and EAG groups were derived through ANOVA (*P* < 0.05), then adjusted with a Duncan multiple-comparison test. All two-sample analyses were performed using Student's *t*-tests. Graphs were plotted in Prism 6.0 (GraphPad Software, CA, USA).

## Results

### Sequence characteristics and homology analysis of *DarmCSP*s

**Figure 1:**
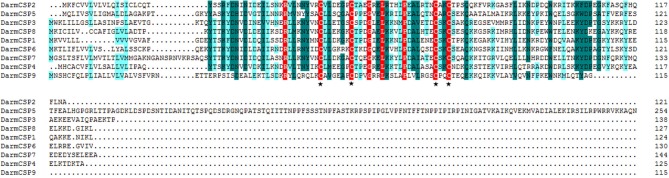
Alignment of amino acid sequences of DarmCSPs. The predicted signal peptides were boxed and four conserved cysteines were labeled with ⋆ below the alignment.

Nine full-length putative *CSP* genes were cloned from *D. armandi*. Generally, *DarmCSP* ORFs contained ~400 nucleotides, encoding ~130 amino acids; the exception was *DarmCSP5* with 786 nucleotides encoding 255 amino acids. Predicted molecular weights of DarmCSPs were 13.02–16.41 kDa, apart from DarmCSP5 at 28.04 kDa. Isoelectric points of DarmCSPs ranged from 4.92 to 9.48, with DarmCSP3 and DarmCSP7 being <7.00 and the remainder >7.00 (Table 1). Nine DarmCSPs contained a putative signal peptide at the N-terminus (Table 1, Figure 1).

Full-length BLASTp searches indicated high amino acid sequence identity between DarmCSPs and CSPs of other bark beetle species. DarmCSP5 showed 79% identity with *Dendroctonus valens* CSP4, whereas other DarmCSPs shared 86–97% identity with *Dendroctonus ponderosae* CSPs (Table 1). DarmCSP amino acid sequence alignment revealed a typical four-cysteine motif at conserved positions. In addition, nine DarmCSPs shared four conserved amino acids: one arginine before the first “C,” as well as one glycine, one leucine, and one proline between the second and third “C.” DarmCSP5 contained an exceptionally long C-terminus (Figure 1).

**Figure 2:**
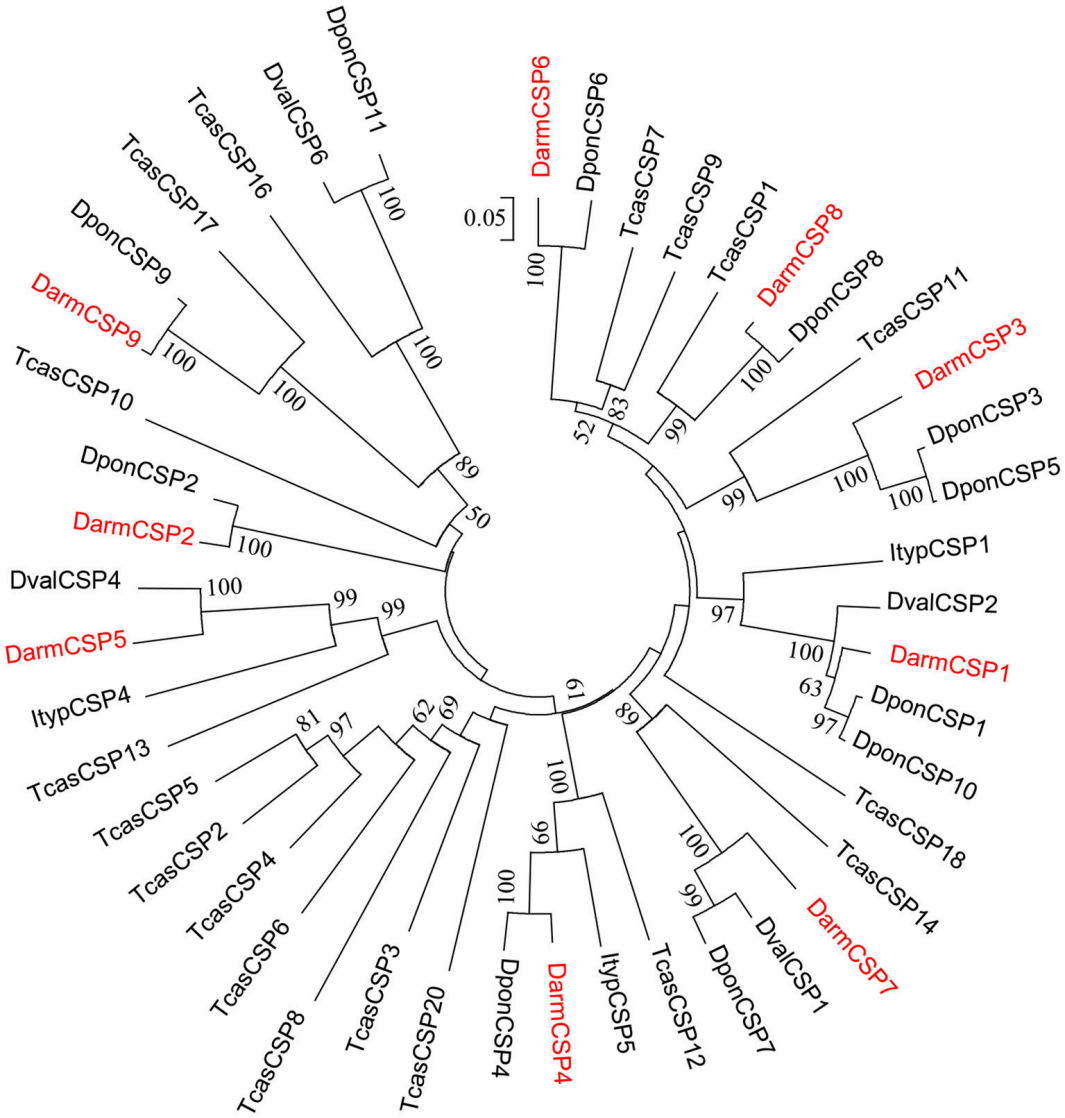
Phylogenetic tree of putative CSPs from *Dendroctonus armandi* (Darm), *Dendroctonus ponderosae* (Dpon), *Dendroctonus valens* (Dval), *Ips typographus* (Ityp), and *Tribolium castaneum* (Tcas). The *D. armandi* translated unigenes were shown in red. The tree was constructed with MEGA6.0, using the neighbor-joining method. Values indicated at the nodes are bootstrap values based on 1000 replicates, and the bootstrap values below 50% are not shown.

Phylogenetic analysis indicated that DarmCSPs were clustered together with CSPs of other bark beetles (*D. ponderosae, Ips typographus*, and *D. valens*). However, DarmCSPs were divergent in both the phylogenetic and homology trees, with only 25–56% amino acid identity within species (Figure 2, Figure S1).

### Distribution of *DarmCSPs* across development and tissues

#### Expression patterns across development

**Figure 3:**
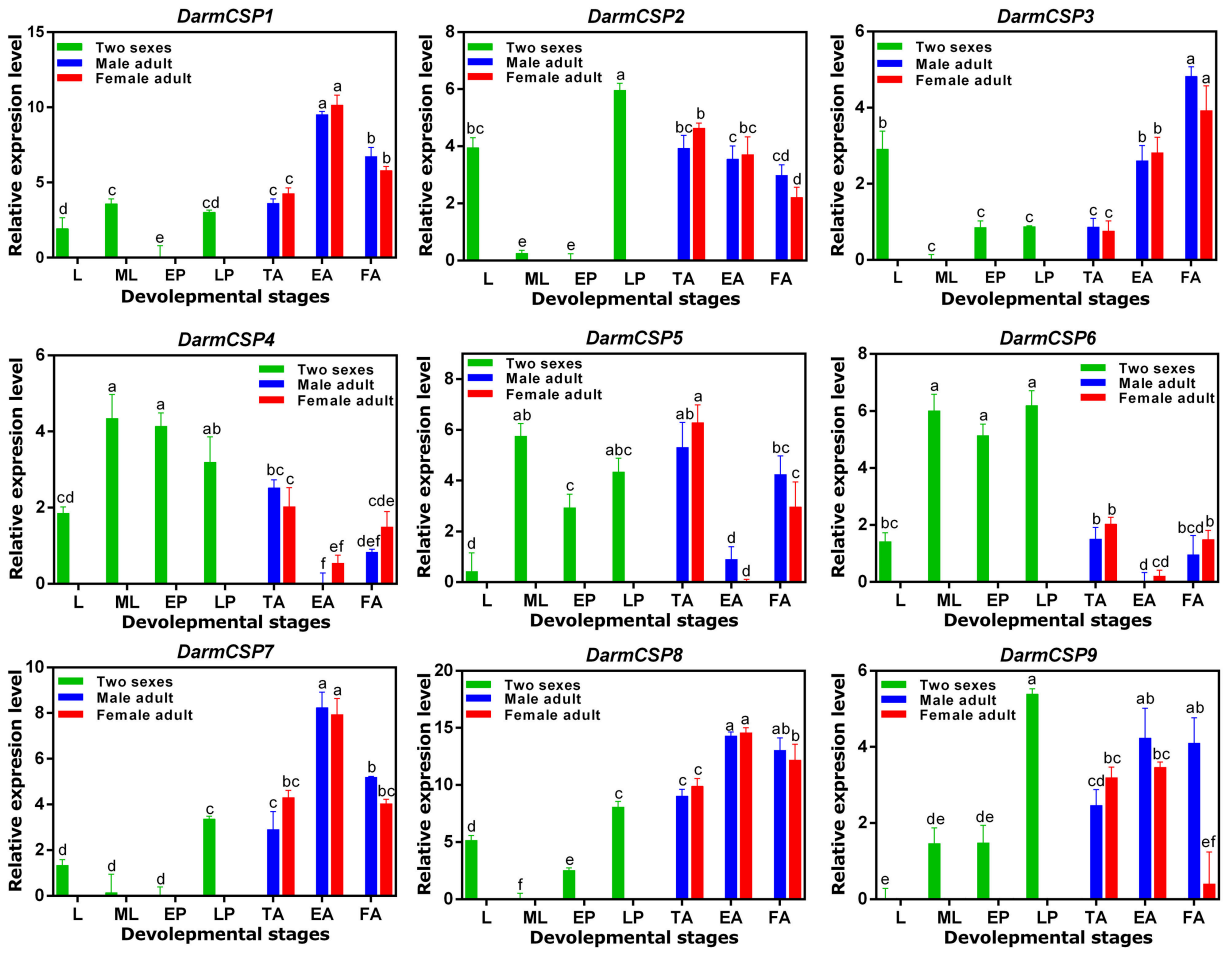
Relative mRNA expression levels of the *DarmCSPs* in different developmental stages. The 2^−ΔΔCt^ values were log2-transformed for analysis of variance and plotting. L, larvae; ML, mature larvae; EP, early stage pupae; LP, late stage pupae; TA, teneral adult; EA, emerged adult; FA, feeding adult. The significant differences between different stages of *DarmCSPs* were marked with letters (*P* < 0.05, one-way ANOVA). All values are mean ± sd, *n* = 3.

*DarmCSP*s were broadly expressed across development of *D. armandi*, but with different profiles. Interestingly, *DarmCSP1, DarmCSP3, DarmCSP7*, and *DarmCSP8* were highly expressed in adults, but had significantly lower expression in larvae and pupae. *DarmCSP1, DarmCSP7*, and *DarmCSP8* were highly expressed in emerged adults, whereas *DarmCSP3* was highly expressed in feeding adults. In contrast, *DarmCSP4, DarmCSP5*, and *DarmCSP6* were highly expressed in mature larvae and pupae, but lowly expressed in adults, especially at the emerged sub-stage. *DarmCSP2* and *DarmCSP9* were more highly expressed during the late pupae stage than in other stages, but their expression was also relatively high in adults. *DarmCSP2* and *DarmCSP3* had relatively high expression in larvae only (Figure 3).

#### Expression patterns across tissues

**Figure 4:**
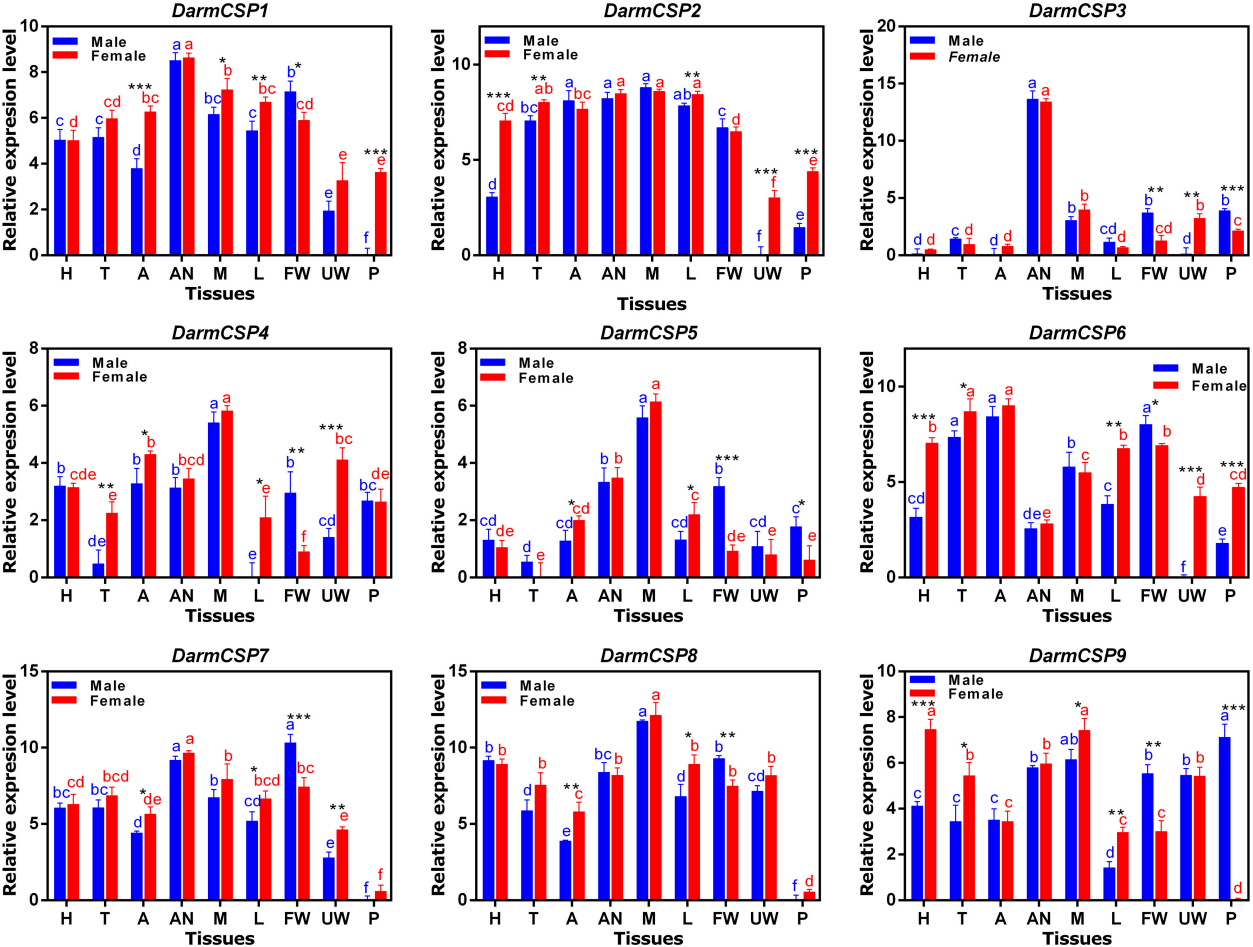
Relative mRNA expression levels of the *DarmCSPs* in different tissues of emerged adults. The 2^−ΔΔCt^ values were log2-transformed for analysis of variance and plotting. H, head (without antenna and mouthpart); T, thorax; A, abdomen (without pheromone gland); AN, antenna; M, mouthpart; L, leg; FW, forewing; UW: underwing; P, pheromone gland. The significant differences between different tissues of female are marked with red letters and male are marked with blue letters (*P* < 0.05, one-way ANOVA). The asterisk indicates a significant difference between female and male expression levels (*P ≤ 0.05, **P ≤ 0.01, ***P ≤ 0.001, Independent-Samples *T*-Test). All values are mean ± sd, *n* = 3.

Nine *DarmCSP*s were expressed at varying levels and with occasional sex differences across multiple tissues. *DarmCSP1, DarmCSP2, DarmCSP3*, and *DarmCSP7* were highly expressed in antennae of both sexes. *DarmCSP3* expression was predominantly in this tissue, but the remaining three were also ubiquitous in other tissues at relatively high levels. Specifically, *DarmCSP2* was highly expressed in mouthparts, abdomens, thoraxes, and legs, with a significantly higher expression in females than in males among the latter two tissues. *DarmCSP7* was more highly expressed in male than in female forewings. *DarmCSP4, DarmCSP5*, and *DarmCSP8* had significantly higher expression in both male and female mouthparts, whereas *DarmCSP9* expression was significantly higher in female mouthparts. *DarmCSP9* was also more highly expressed in female than in male heads. However, its expression was significantly higher in male pheromone glands. Apart from its high expression in mouthparts, *DarmCSP8* was also present in other tissues at relatively high levels. Finally, *DarmCSP6* was ubiquitous in most tissues, with notably high expression in abdomens and thoraxes but low expression in antennae (Figure 4).

### Binding characteristics of DarmCSPs

#### Bacterial expression and purification of DarmCSPs

Three pET32a(+)/DarmCSPs were successfully induced and expressed in BL21(DE3) PlysS cells. DarmCSP1 and DarmCSP2 exhibited good yield (more than 20 mg/L), whereas DarmCSP3 had lower expression. These three proteins were located in the supernatant after sonication. The results of 12% SDS-PAGE indicated that recombinant and pure proteins without His-tags were respectively present as single bands at 32.0 and 14.0 kDa (without signal peptide) (Figure S2). This outcome accords with deduced molecular weights of the predicted amino acid sequences.

#### Fluorescence binding assays of DarmCSPs

**Figure 5:**
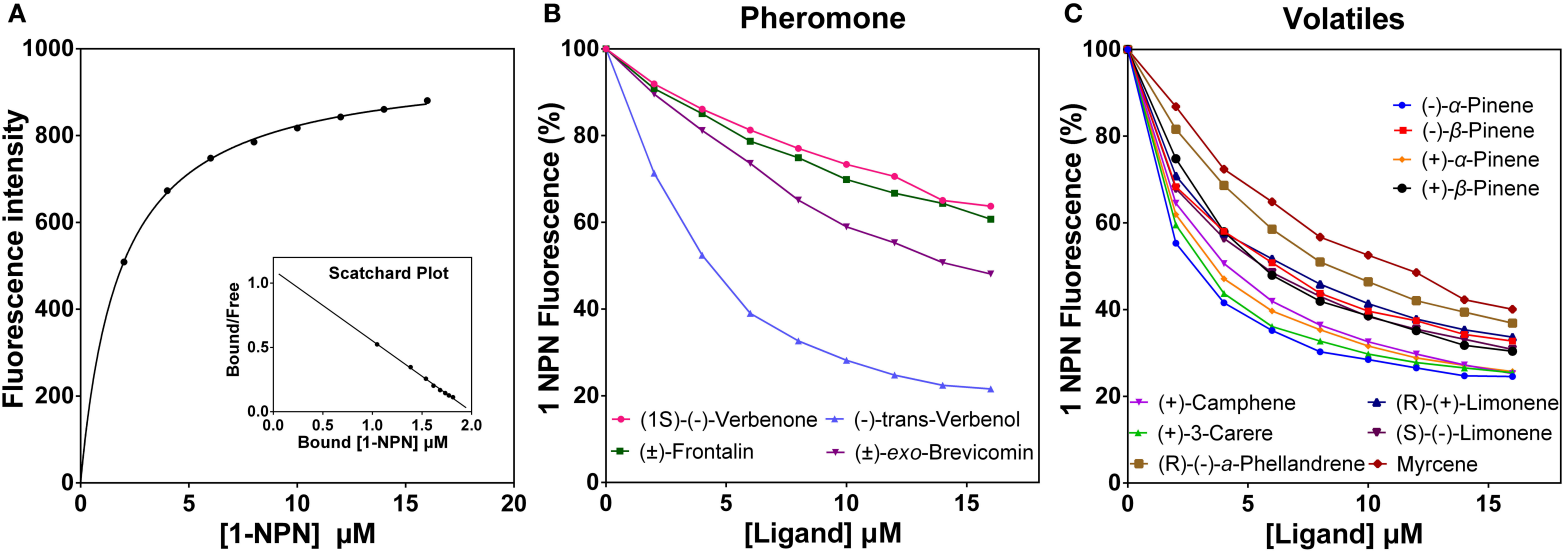
Fluorescent competition binding assay of DarmCSP2. **(A)** Binding curve of 1-NPN with puried DarmCSP2 and its relative Scatchard plot (inset). Dissociation constant of 1-NPN with DarmCSP2 is 1.84 ± 0.04 μM. **(B,C)** Competitive binding curves of the DarmCSP2 to volatile compounds (see Table 2). **(B)** Four pheromones components of *D. armandi*; **(C)** Ten host volatiles of *P. armandi*.

DarmCSP2 interacted strongly with 1-NPN, exhibiting dissociation constants of 1.84 ± 0.04 μM. In contrast, DarmCSP1 and DarmCSP3 had no obvious affinity to 1-NPN. Saturation results and linear Scatchard plots revealed only a single binding site for 1-NPN in DarmCSP2, with no allosteric effects, indicating that 1-NPN was suitable as the fluorescence probe (Figure 5A).

Fluorescence competitive binding assays revealed high binding affinity (Ki < 10 μM) of DarmCSP2 to all tested host volatiles, especially (−)-α-pinene and (+)-3-carene (Ki = 1.64 ± 0.08 μM and Ki = 1.97 ± 0.46 μM, respectively) (Figure 5B, Table 2). Notably, DarmCSP2 showed high (Ki < 10 μM) and moderate affinity (Ki < 20 μM) to four pheromones (two in each category), with especially strong bonds to (−)-trans-verbenol (Ki = 2.80 ± 0.07 μM) (Figure 5C, Table 2).

#### Structural model of DarmCSP2

**Figure 6:**
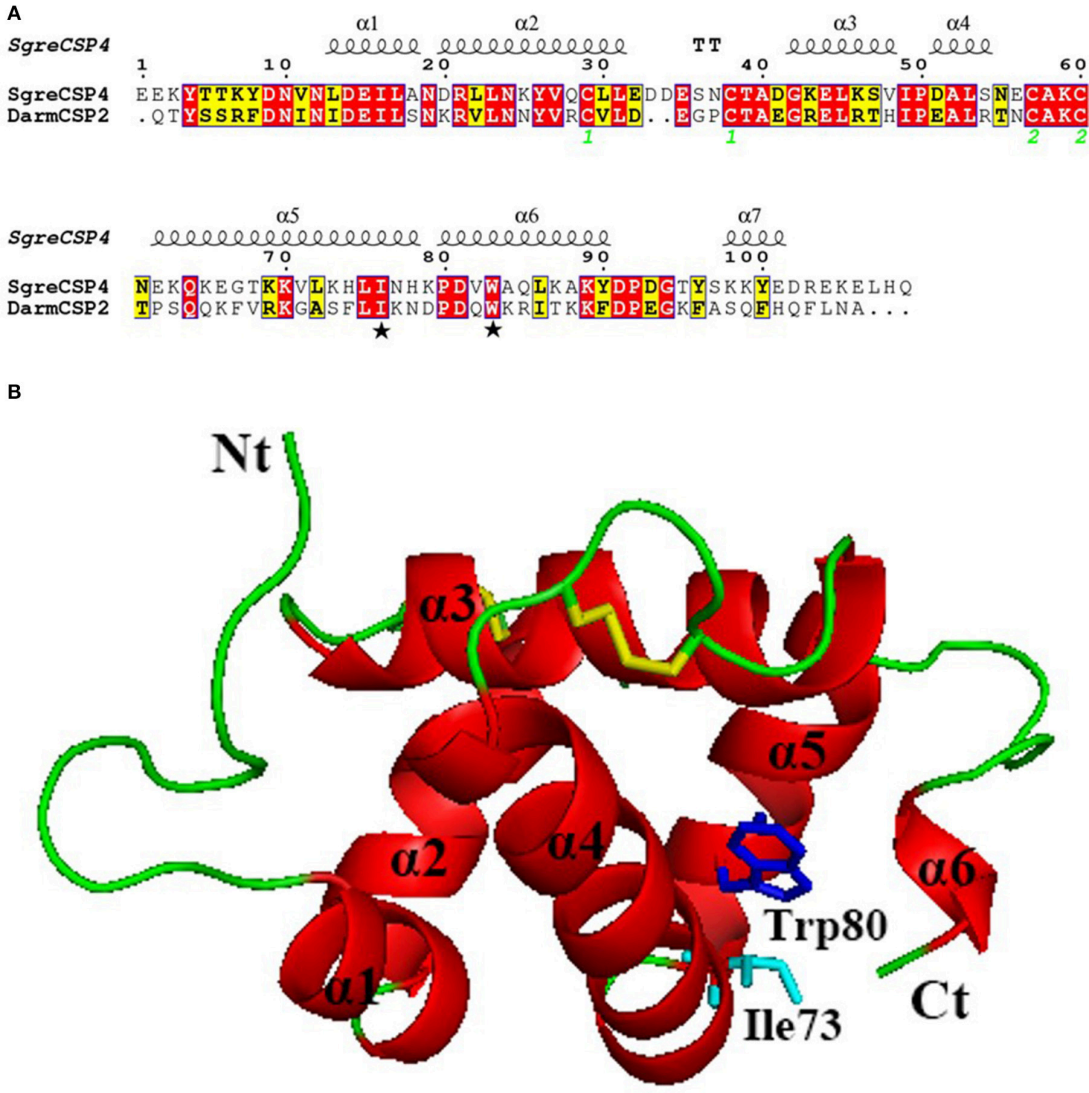
Three-dimensional model of DarmCSP2**. (A)** Sequence alignment of DarmCSP2 and CSP4 from *S. gregaria*. Conserved residues are highlighted in white letters with a red background. Alignment positions are framed in blue if the corresponding residues are identical or similar. Ile 73 and Trp 80 are labeled with pentagrams. The disulfide bridges are numbered 1 and 2. **(B)** Overall structural model of DarmCSP2. Six α- helices are labeled in red. Residues of Ile 73 and Trp 80 are shown as stick, colored in light blue and blue respectively. Disulphide bridges are colored yellow.

The 3D-structural model of DarmCSP2 revealed six α-helices, plus a very short one near the carboxyl terminus, all connected with loops to form a binding pocket. This structure is typical of CSPs. Active sites I73 and W80 in DarmCSP2 corresponded to I76 and W83 residues in *S. gregaria* CSP4 (Figures 6A,B).

### Efficiency analysis of RNAi on *DarmCSP2*

#### Effect of dsRNA treatment on *DarmCSP2* transcript level

Injection of dsCSP2 significantly decreased target gene expression level, according to qRT-PCR results. The dsCSP2-injected group did not differ from controls (non-injected and water-injected) in *DarmCSP2* mRNA levels 12 h post-injection, a significant difference emerged after 24 h, followed by a continuous decrease from control levels after 48 h (Figure S3).

#### Effect of dsRNA treatment on electrophysiological responses to host volatiles and pheromones

**Figure 7:**
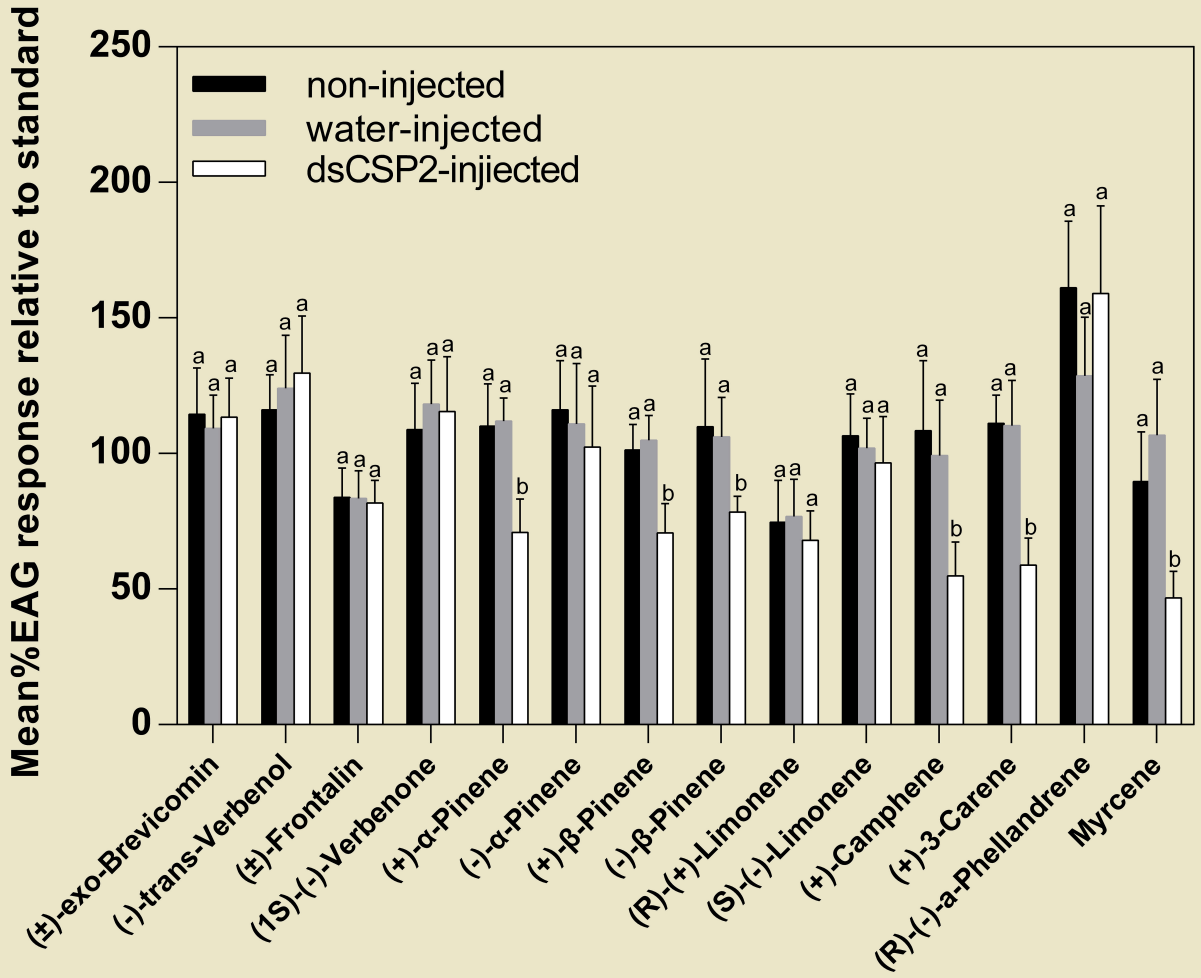
Effect of RNAi on EAG responses of *D. armandi* to volatile compounds (see Table 2). Letters on the bar are the significant difference among different treatments in the same volatile compounds (*P* < 0.05, one-way ANOVA). All values are mean ± sd, *n* = 3.

At 48 h post-injection, dsCSP2-injected antennae did not exhibit significant decreases in response to four test pheromones, compared with controls. However, dsCSP2 injection significantly reduced antennae EAG activity in response to six test host volatiles, including: (+)-α-pinene, (+)-β-pinene, (−)-β-pinene, (+)-camphene, (+)-3-carene, and myrcene (Figure 7).

## Discussion

In this study, nine full-length *DarmCSP* genes were cloned and identified. This number is close to the amount found in several other bark beetle species: 11 in *D ponderosae* (Andersson et al., 2013), six in *D. valens* (Gu et al., 2015), and six in *I. typographus* (Andersson et al., 2013). *DarmCSPs* were classical *CSP* genes based on a variety of hallmarks (Vieira and Rozas, 2011). First, their deduced amino acid sequence revealed a typical four-cysteine motif at conserved positions, conforming to the CSP model of C1-X6–8-C2-X16–21-C3-X2-C4 (X represents any amino acid) (Pelosi et al., 2006). Furthermore, at the N-terminus, DarmCSPs contained a putative signal peptide of 16–25 amino acids in length (Figure 1).

DarmCSPs were closely related to CSPs in other bark beetles. Exhibiting high amino acid sequence identity with *D. ponderosae* and *D. valens* CSPs (Table 1), DarmCSPs were also clustered together with CSPs of other bark beetles in the phylogenetic analysis (Figure 2). Previous reports have indicated that *D. ponderosae, I. typographus, and D. valens CSP* genes are orthologous (Andersson et al., 2013; Gu et al., 2015). Together, these results indicated that bark-beetle *CSP* genes may have similar expression profiles and function.

Among the nine DarmCSPs, amino acid sequences exhibited considerable variation in identity similarity (25–56%) (Figure S1). This variation was similar to sequence identity percentages in *Nilaparvata lugens* (10–77%) (Yang et al., 2014), *Bombyx mori* (10–50%) (Qiao et al., 2013), and *Papilio xuthus* (20–70%) (Ozaki et al., 2008). Reflecting the sequence variation, all nine DarmCSPs were distributed in different branches of the phylogenetic tree, a pattern also found in other bark beetles (Andersson et al., 2013; Gu et al., 2015). The diversification in DarmCSP amino acid sequences suggested multiple functions.

Supporting that idea is the observation of broad variety in expression patterns among *DarmCSP*s (Figures 3, 4). *DarmCSP4, DarmCSP5*, and *DarmCSP6* were all highly expressed in mature larvae and pupae, stages when insects stop feeding and experience enormous morphological changes. We also observed a sudden upregulation of *DarmCSP2* and *DarmCSP9* before emergence. Therefore, these five *DarmCSP* genes may be involved in *D. armandi* metamorphosis. Findings in other insects support this conclusion. Specifically, regulation of *CSP* expression in *Choristoneura fumiferana, B. mori*, and *Nilaparvata lugens* varies with hormonal changes during metamorphosis (larvae and pupae or nymphs) (Wanner et al., 2005; Gong et al., 2007; Yang et al., 2014; Hou et al., 2016).

*DarmCSP1, DarmCSP2, DarmCSP3*, and *DarmCSP7* were highly expressed in antennae, with *CSP3* almost exclusively found there. Furthermore, *DarmCSP2, DarmCSP4, DarmCSP5, DarmCSP8*, and *DarmCSP9* genes were enriched in mouthparts. Antennae and mouthparts are the primary chemosensory organs of insects, each covering a different function. The antenna-preferential genes are probably involved in recognizing sex pheromones and plant volatiles (Tomaselli et al., 2006; Qiao et al., 2013; Yang et al., 2014; Li et al., 2015, 2016), whereas mouthpart-preferential genes likely play roles in gustation, recognizing non-volatile food sources or detecting close-range odors (Nagnan-Le Meillour et al., 2000; Jin et al., 2006; de la Paz Celorio-Mancera et al., 2012; Hua et al., 2012).

We also found that *DarmCSP1, DarmCSP2, DarmCSP7, DarmCSP8*, and *DarmCSP9* were ubiquitous in other tissues at relatively high levels, suggesting involvement in other adult physiological processes (Nomura et al., 1992; Gong et al., 2012; Gu et al., 2013; Zhou et al., 2013). In particular, *DarmCSP6* was ubiquitous and highly expressed in most tissues, especially the abdomens and thoraxes. Coupled with its relatively low expression in antennae, these results suggest that *DarmCSP6* mainly affects physiological processes, but not excluding chemoreception. In sum, tissue and developmental expression profiles indicate that *DarmCSPs* serve numerous functions in metamorphosis, olfaction, and gustation.

Because the primary mechanism of insect CSPs is to recognize and bind exogenous hydrophobic chemicals to receptors through the sensillum lymph of chemosensory organs (Pelosi et al., 2005; Liu et al., 2012; Leal, 2013), we examined the binding affinity of DarmCSPs. Our binding assays revealed that DarmCSP2, but not DarmCSP1 or 3, has high affinity for 1-NPN, partially corresponding to *B. mori* data showing that BmorCSP1 and 2 bound well to 1-NPN, whereas BmorCSP9 and 12 do not (Qiao et al., 2013). Furthermore, when we examined the competitive ligand binding properties of DarmCSP2 specifically, we found that the protein bound strongly to all tested host volatiles [especially (−)-α-pinene and (+)-3-carene] and various pheromones [especially to (−)-trans-verbenol]. In previous studies, the tested volatiles effectively elicited different degrees of EAG responses in *D. armandi* antennae, and some of them were an important constituent of attractants of *D. armandi* (Zhang et al., 2010; Xie and Lv, 2012; Chen et al., 2015; Zhao et al., 2017a,b).

The 3D model of DarmCSP2 revealed an internal hydrophobic binding cavity formed from six α-helices, corresponding to existing studies on CSP structure (Campanacci et al., 2003; Mosbah et al., 2003; Tomaselli et al., 2006; Kulmuni and Havukainen, 2013). Additionally, active sites I73 and W80 in DarmCSP2 corresponded to I76 and W83 residues in SgerCSP4, confirmed to bind oleamide (Tomaselli et al., 2006). Thus, these active sites are likely involved in binding to pheromones. Combined with the high expression of DarmCSP2 in antennae and mouthparts, these data suggest that DarmCSP2 may be a major carrier of the tested ten host volatiles and four pheromones of *D. armandi*. Data on CSPs in diverse insects also support this binding function: the proteins bind pheromone components in *Schistocerca gregaria* (Li et al., 2015), host plant volatiles and non-volatile secondary metabolites in *Apolygus lucorum* (Hua et al., 2012), as well as host plant volatiles and sex pheromones in *Sesamia inferens* and *Microplitis mediator* (Zhang et al., 2014; Peng et al., 2017).

The importance of DarmCSP2 in binding to major volatiles was further confirmed by our RNAi experiment. The injection of dsCSP2 significantly decreased *DarmCSP2* expression, and antennae subjected to RNAi experienced significantly reduced EAG activity in response to six tested host volatiles [(+)-α-pinene, (+)-β-pinene, (−)-β-pinene, (+)-camphene, (+)-3-carene, and myrcene], but not in response to pheromones. This list corresponded well with the list of volatiles found to be bound by DarmCSP2 in fluorescence binding assays. Our results corroborate recent RNAi studies that demonstrated how the silencing of genes encoding OBPs or CSPs abolished or modified electrophysiological responses, influenced odor preferences, disrupted behavior, and altered morphology in insects (Maleszka et al., 2007; Gong et al., 2012; Yi et al., 2013; Wu et al., 2016; Dong et al., 2017; Zhang et al., 2017). Together, our results and previous work suggest that DarmCSP2 collaborates with multiple binding proteins (including other CSPs and OBPs) to transport numerous compounds. For instance, in *Anopheles gambiae*, OBP1 and OBP4 were co-expressed in some antennal sensilla, forming heterodimers in the sensillum lymph that differed in binding characteristics from the individual proteins (Qiao et al., 2011). In *Adelphocoris lineolatus*, a mixture of AlinCSP5 and AlinCSP6 increased binding affinities to terpenoids that did not bind with individual AlinCSP (Sun et al., 2015). In *Helicoverpa armigera*, HarmPBP1 and HarmPBP2 were associated with the recognition of the major sex pheromone component, Z11-16:Ald (Dong et al., 2017). Indeed, this phenomenon of olfaction-related binding proteins forming complexes may be universal across insects, given the clear advantages in increasing binding capacity and accuracy, thus expanding their chemical communication potential.

In this study, we combined molecular and physiological methods to clarify DarmCSPs characteristics and functions. We hypothesized that they are involved in developmental metamorphosis, as well as olfaction and gustation in the adult chemosensory system. Their role in olfaction was particularly notable; CSP2 was abundant in antennae and carried host volatiles that regulated *D. armandi* foraging behaviors. These data clarified the molecular mechanisms of olfactory perception in *D. armandi*, providing a theoretical foundation for eco-friendly pest control.

## Author contributions

ZL, LD, and HuC designed the experiments. ZL, HoC, DF, and YS preformed the experiments; ZL analyzed data and drafted manuscript. ZL, LD, HoC, and HuC revised the manuscript. All authors read and approved manuscript for final submission.

### Conflict of interest statement

The authors declare that the research was conducted in the absence of any commercial or financial relationships that could be construed as a potential conflict of interest.

## Supplementary material

Figure S1 Homology Tree of amino acid of DarmCSPs. The tree was constructed with DANMAN. The percentages were the identity of DarmCSPs.

Figure S2 Prokaryotic expression and purification of three CSPs of *D. armandi* analyzed on SDS-PAGE (12%). Lane M, protein molecular weight marker (top to bottom: 200, 66.4, 44.3, 29.0, 20.1, 14.3, 6.5 kDa); Lane 1, total protein extracted from BL21 bacteria cells with pET32a/DarmCSP vector after induced by IPTG; Lane 2, protein in precipitate after sonication; Lane 3, protein in supernatant after sonication; Lane 4, purified fusion protein pET32a(+)/CSP; Lane 5, purified DarmCSP after His-tag cleavage by rEK.

Figure S3 Relative expression level of *DarmCSP2* at 12, 24 and 48 h after dsCSP2 injection. Non-injected and water-injected were as control. The significant differences among different treatments in every time point were marked with letters (*P* < 0.05, one-way ANOVA, all values are mean ± sd, *n* = 3).

Table S1 Primers used for gene isolation, RT-qPCR, Prokaryotic expression, and dsRNA synthesis.
