# Supplementary figures and images for: Identification, Expression Patterns, and Functional Characterization of Chemosensory Proteins in Dendroctonus armandi (Coleoptera: Curculionidae: Scolytinae)
Source: Front Physiol. 2018 Mar 27;9:291. doi: 10.3389/fphys.2018.00291 (PMC5881420; doi:10.3389/fphys.2018.00291)

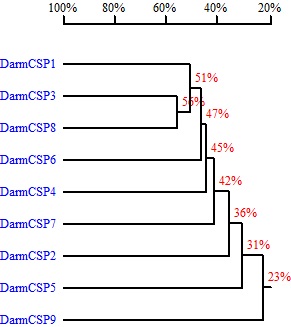

Supplement: Figure S1 — Homology Tree of amino acid of DarmCSPs. The tree was constructed with DANMAN. The percentages were the identity of DarmCSPs. [file Image1.JPEG]

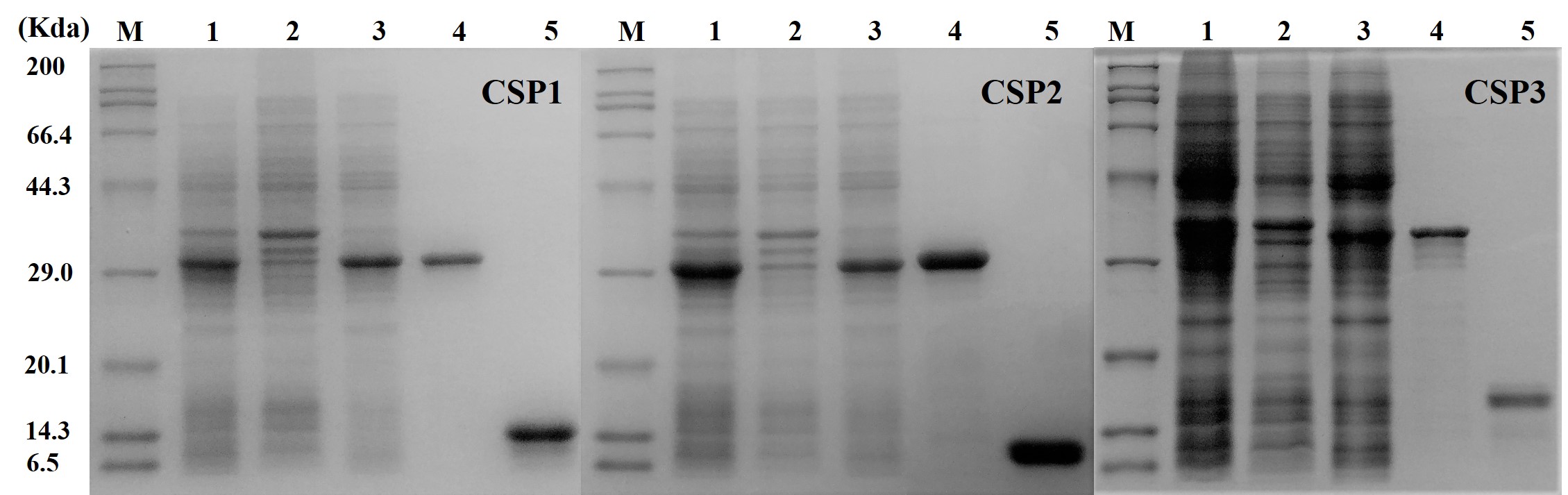

Supplement: Figure S2 — Prokaryotic expression and purification of three CSPs of D. armandi analyzed on SDS-PAGE (12%). Lane M, protein molecular weight marker (top to bottom: 200, 66.4, 44.3, 29.0, 20.1, 14.3, 6.5 kDa); Lane 1, total protein extracted from BL21 bacteria cells with pET32a/DarmCSP vector after induced by IPTG; Lane 2, protein in precipitate after sonication; Lane 3, protein in supernatant after sonication; Lane 4, purified fusion protein pET32a(+)/CSP; Lane 5, purified DarmCSP after His-tag cleavage by rEK. [file Image2.JPEG]
