# Supplementary material for: Identification, Expression Patterns, and Functional Characterization of Chemosensory Proteins in Dendroctonus armandi (Coleoptera: Curculionidae: Scolytinae)
Source: Front Physiol. 2018 Mar 27;9:291. doi: 10.3389/fphys.2018.00291 (PMC5881420; doi:10.3389/fphys.2018.00291)
Supplement: Table S1 — Primers used for gene isolation, RT-qPCR, Prokaryotic expression, and dsRNA synthesis. [file Table1.DOCX]

**Supplementary Materials**

**Table S1. Primers used for gene isolation, RT-qPCR, Prokaryotic expression and dsRNA synthesis.**

| **Purpose** | **Primer sequence (5’-3’)** | |
| --- | --- | --- |
| **cDNA amplification** | **Forward** | **Reverse** |
| DarmCSP1 | CATAACGATCGACTGATGAGAAACtayrtnraytg | CTGGTACTTCTTCTTGTAAGTTCCTTCTkkrtcrtaytt |
| DarmCSP2 | TCTTGCAAATTGCCATTTGCT | ATTGATCGGGGTCGTTTTTGA |
| DarmCSP3 | TTCTTCTCGGCTCACTCCTC | TCCAATACTCCGGCTTGTTG |
| DarmCSP4 | AGCCCGTACACCAGCAAAT | GCTCAATCGGTCCCAATCT |
| DarmCSP5 | GGATGGCTTTGGACCTGAC | GGGGATGGGCGGATTAGTA |
| DarmCSP6 | AAGACATTACCATCTCCCCTC | GCAACTCATTGAACCATCCAC |
| DarmCSP7 | GTCCTCACGGTGCTGATGAT | GCTAACTGGGGTGCTGTTTT |
| DarmCSP8 | CATAACGATCGACTGATGAGAAACtayrtnraytg | CTGGTACTTCTTCTTGTAAGTTCCTTCTkkrtcrtaytt |
| DarmCSP9 | TGAAACAACGGAAAGACCA | TCGCAATCAACCTGCATAG |
| **3’ RACE** | **Inner** | **Outer** |
| DarmCSP1 | AGGAAGGAGCGAAGAAAGTGA | TCTAAATACACTCCCAAGCAA |
| DarmCSP2 | ATTGCGCCAAATGTACGCCCTCT | CTTGGATGAGGGTCCATGTACTGC |
| DarmCSP3 | GCCTGCTGGAAACGGGCTCTTG | ACGAGCGTCTGCTGAAGAACTA |
| DarmCSP4 | ACGACAAACAGAAATCCACTACTGAGAA | GTTGAAAAAGACTCTGCCCGATGC |
| DarmCSP5 | AAGCACTTTTGAAGCCCTCCAT | GATTTGGCAAGAGTTGAGCGGG |
| DarmCSP6 | AGTTCACTTGCCTGACGCTCTAC | TTGATTGTGTGTTGGGGAAAAGG |
| DarmCSP7 | CAACTGTTTGCTGGACAAGGGC | GGGTGAAGCGAAGTGCCCAAAC |
| DarmCSP8 | AACAAGCCAGAATGGTGGACAG | GGGCAAATGTACTCCCGATGGT |
| DarmCSP9 | GCTGAAATGTGCAGTGGGCGA | GAATGGAACAAAATGCTACAAAC |
| **5’ RACE** | **Inner** | **Outer** |
| DarmCSP1 | TAATGAATCACTTTCTTCGCTCC | CTTGATCCCAGTAGTCGCGTTTG |
| DarmCSP2 | GGACCCTCATCCAAGACACACCGAAC | AAACTGGCTCCTTTTCGCACA |
| DarmCSP3 | CAGGAACCTCATCATAACCTCAGA | TGCCAGTGGGGTCATACTTTTCCT |
| DarmCSP4 | CCAGCAAATACGACAATGTAGACG | CACCTGAAGGAAGAGAGTTGAAAAAGAC |
| DarmCSP5 | CCATCAAGGCTGTGGCGGC | GGGTCCCATTTCCCGCTCA |
| DarmCSP6 | GTCCGACTGTAGAGCGTCAGGCA | GATGATTGATGATTTTTCTGCTG |
| DarmCSP7 | GTGAGCAACTCCCAGTCGTCCC | CTGGGGTGCTGTTTTTATGGGA |
| DarmCSP8 | CTGGCTTGTTGTCGATCAAGTGTCG | TTCTGTCCACCATTCTGGCTTGT |
| DarmCSP9 | CAAAGCTTCGTCTGATATGGA | GTCGCCCGACTGGATCACATGG |
| **Full-length verification** | **Forward** | **Reverse** |
| DarmCSP1 | GCATGAAAGTCGTGTTGCTTC | GCTTACAATTTGATATTTTCT |
| DarmCSP2 | GCATGAAATTCTGCGTGGTTC | GCTTAGGCGTTCAGGAATTGG |
| DarmCSP3 | GCATGTGGAAACTAATTCTTC | GCTTAAGGCGTTTTTTCTGCA |
| DarmCSP4 | GCATGCATTGCGCGTGTGTTT | GCCTAGGCAGTTTTGTCAGTC |
| DarmCSP5 | GCATGCAGCTGATTGTTTCGG | GCTCACACATTTTGTGCCTTC |
| DarmCSP6 | GCATGAAAACTTTCATTTTTC | GCTTAAACGATAACTCCCTCT |
| DarmCSP7 | GCATGGGTTCTTTAACGAGCT | GCCTATGCTTCCTCTAGTTCC |
| DarmCSP8 | GCATGAAGATTTGTATTCTTG | GCTTACAATTTAATGCCGTCC |
| DarmCSP9 | GCATGAATTCCCATTGCTTTC | GCTCAACCTGCATAAGTTTGT |
| **RT-qPCR** | **Forward** | **Reverse** |
| DarmCSP1 | CCTGATGGTATTGAACTGA | CCTGCTCCTCATATTTCTT |
| DarmCSP2 | GGAAGAGGATTACGAAGAA | CAAAGCAGCGAAAGTTAT |
| DarmCSP3 | AATACACAACCAAATACGACAA | AACCTCATCATAACCTCAGAA |
| DarmCSP4 | ATCGTGTGCTGACCAACTA | TGCTTGTAGACGCCTTCA |
| DarmCSP5 | CCATCGACGCAAACATCACC | GCCTTCACCGTAGCTCCAAT |
| DarmCSP6 | AATCATCAATCATCTACTG | ATAACTCCCTCTCTTCTA |
| DarmCSP7 | CTGAATGCTCCAAATGTA | GTTCCTATGCTTCCTCTA |
| DarmCSP8 | GGAGCCTACAAGAAGCAATA | CCAGCAGTATTTATTTCCAAGT |
| DarmCSP9 | CACGGAACAAGAGAAGAA | ATACATTAGGGTCATCAATAAAC |
| β-actin | CATCAGGAAGGACTTGTA | GATTCGTCGTATTCTTGTT |
| α-Tubulin | CTGATCTTCCACTCCTTC | GCCTCATTGTCAACCATA |
| **Prokaryotic expression** | **Forward** | **Reverse** |
| DarmCSP1 | CGCGGATCCGATGAATATACCTCAAAATTCGAC | CCGCTCGAGTTACAATTTGATATTTTCTTTTTTGG |
| DarmCSP2 | CGCGGATCCCAAACGTACTCGTCGAGATTT | CCGCTCGAGTTAGGCGTTCAGGAATTGG |
| DarmCSP3 | CGCGGATCCGAAGTGACCGGAAAAACTCAA | CCGCTCGAGTTAAGGCGTTTTTTCTGCAGG |
| **dsRNA synthesis** | **Forward** | **Reverse** |
| T7DarmCSP2 | GGATCCtaatacgactcactataggCGTGGTTCTGGTGTTGGT | GGATCCtaatacgactcactataggGGCGCAATTCGTTCTCAA |
| DarmCSP2 | CGTGGTTCTGGTGTTGGT | GGCGCAATTCGTTCTCAA |

Note: GGATCC is restriction enzyme cutting site of BamHⅠ; CTCGAG is restriction enzyme cutting site of XhoⅠ; CGC and CCG before prokaryotic expression prime is protective bases; “taatacgactcactatagg” is the minimal T7 RNA polymerase promoter sequence requirement; “GGATCC” : the addition of extra bases upstream of the minimal T7 RNA polymerase promoter sequence may increase yield by allowing more efficient polymerase binding and initiation.
